# Macronutrient balance and lifespan

**DOI:** 10.18632/aging.100098

**Published:** 2009-10-22

**Authors:** Stephen J. Simpson, David Raubenheimer

**Affiliations:** ^1^ School of Biological Sciences, The University of Sydney, NSW 2006, Australia; ^2^ Institute of Natural Sciences, Massey University, Albany, New Zealand

**Keywords:** caloric restriction, dietary restriction, protein, geometric framework, nutrient balance, TOR, AMPK

## Abstract

Dietary restriction (DR) without malnutrition is widely regarded to be a universal mechanism for prolonging lifespan. It is generally believed that the benefits of DR arise from eating fewer calories (termed caloric restriction, CR). Here we argue that, rather than calories, the key determinant of the relationship between diet and longevity is the balance of protein to non-protein energy ingested. This ratio affects not only lifespan, but also total energy intake, metabolism, immunity and the likelihood of developing obesity and associated metabolic disorders. Among various possible mechanisms linking macronutrient balance to lifespan, the nexus between the TOR and AMPK signaling pathways is emerging as a central coordinator.

Convincingly separating the effects of CR on lifespan from more specific nutrient effects is not trivial and requires experimental designs comprising multiple dietary regimes in which energy intake and nutrient balance are considered both separately and interactively [1]. Building upon an earlier study questioning the role of CR in *Drosophila melanogaster* [2], the first study to employ a design that unequivocally disentangled CR from specific nutrient effects was that of Lee et al. [3]. Mated female flies were allowed ad libitum access to one of 28 diets, varying in the ratio and concentration of yeast to sugar. Food intake was measured for each fly and bi-coordinate intakes of protein and carbohydrate (the major macronutrients in the diets) were plotted. Response surfaces for lifespan, age of maximal mortality, rate of age-dependent increase in mortality, lifetime egg production and rate of egg production were then fitted over the array of protein-carbohydrate intake points (see Figure 1A for lifespan and lifetime egg production surfaces). Flies lived longest on a diet containing a 1:16 P:C ratio and lived progressively  less long as the P:C ratio increased. The contours of the longevity surface ran almost orthogonally to lines of equal caloric intake (dotted lines in Figure 1A). Even allowing for possible differences in the relative availability of energy in protein and carbohydrate or interactions between protein and carbohydrate metabolism, the lifespan and caloric intake isoclines in Figure 1A cannot be aligned. The data therefore prove that CR could not account for the variation in lifespan. Rather, the balance of carbohydrate to protein ingested was strongly correlated with longevity.

The response surface for lifetime egg production peaked at a higher protein content than supported maximal lifespan (1:4 P:C, Figure 1A). This demonstrates that the flies could not maximize both lifespan and egg production rate on a single diet, and raises the interesting question of what the flies themselves prioritized - extending lifespan or maximizing lifetime egg production. Lee et al. [3] answered this by offering one of 9 complementary food choices in the form of separate yeast and sugar solutions differing in concentration. The flies mixed a diet such that they converged upon a nutrient intake trajectory of 1:4 P:C, thereby maximizing lifetime egg production and paying the price of a diminished lifespan.

**Figure 1.**
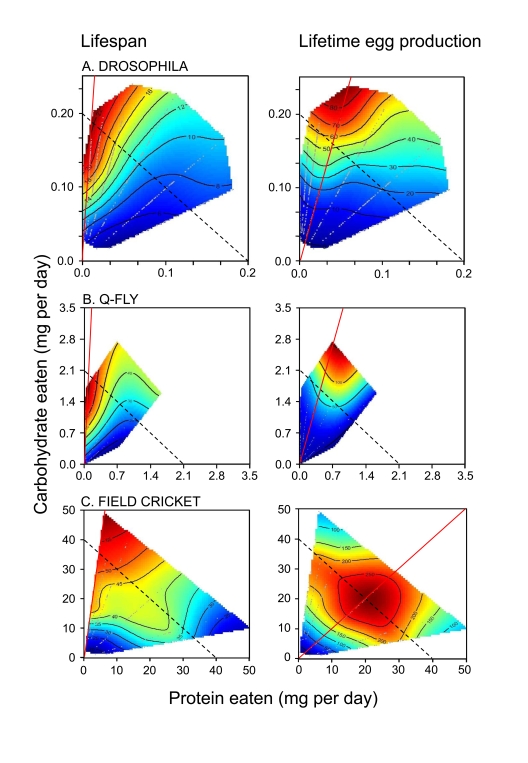
How the intake of protein and carbohydrate influence longevity and lifetime egg production in adults of three insect species. Individuals were given ad libitum access to one of 28 (*Drosophila *and the Queensland fruit fly, Q-fly) or 24 (field cricket) diets varying in the ratio and total concentration of protein to carbohydrate (P:C). Plotted onto arrays of points of nutrient intake are fitted surfaces for the two performance variable, which rise in elevation from dark blue to dark red. Unbroken red lines indicate the dietary P:C that maximized the response variable, whereas the dotted lines indicate isocaloric intakes. In each case, insects lived longest when the diet contained a low P:C, and lifespan declined as P:C rose. Female reproductive output was maximal on higher P:C diets than sustained greatest longevity, but fell as P:C rose further, even at high total energy intakes. Data are replotted from Lee et al. [3] (*Drosophila*), Maklakov et al. [11] (field crickets), and Fanson et al. [4] (Q-fly).

Lee et al. [3] compared their data against a longevity surface compiled from previously published studies, individually involving many fewer dietary treatments and no measurement of long-term food intake. The two surfaces corresponded closely, despite substantial procedural differences across studies and differences in mean lifespan between capillary-fed, singly housed flies in the study of Lee et al. [3] and flies housed in groups and fed agar-based diets in the other experiments. To further demonstrate that the nutritional associations were robust, traditional demography cage trials were run for a selection of diets without measuring intake. These flies lived longer than when housed singly and fed from capillaries, but the pattern of lifespan, egg production and egg production rate in relation to dietary P:C ratio was the same.

A parallel experiment was conducted by Fanson et al. [4] on Queensland fruit fly, *Bactrocera tryoni* (another dipteran but from a different family, Tephritidae rather than Drosophilidae) subjected to one of 28 no-choice or 25 choice diet treatments. As can be seen in Figure 1B, the results and conclusions were similar in all respects to those reported by Lee et al. [3] for *Drosophila*. Once again, dietary P:C and not energy intake was strongly associated with lifespan. The data were also consistent with those from studies on another species of tephritid, the Mexican fruit fly,*Anastrepha ludens* [5].

Recently, Ja et al. [6] confirmed that increasing the ratio of yeast to sugar (hence P:C)  in the diet substantially reduced lifespan in adult *Drosophila*, to an extent that maps precisely onto the data of Lee et al. [3]. Additionally, these authors found that the more modest shortening of lifespan found on concentrated relative to dilute versions of a diet containing a 1:1 yeast to sugar ratio (the diet composition employed in many previous studies) was absent when flies had access to free water; implying that what has previously been reported as the beneficial effects of DR may instead be the obverse of the deleterious consequences of water deprivation. Providing a separate water source had no effect on the change in lifespan associated with a change in yeast:sugar.  Indeed, it can now be suggested with some credence that perhaps the life-prolonging effects of DR, as traditionally conceived, do not occur in *Drosophila*. It is interesting to note how a recent study [7] denotes an increase in P:C combined with overall dilution as ‘diet restriction', rather than relying on dilution of a 1:1 yeast:sugar diet as in the past.

In the studies of Lee et al. [3], Fanson et al. [4], Ja et al. [6] and others, longevity was primarily associated with the ratio of yeast to sugar eaten. Yeast is a complex food, containing micronutrients and other chemicals in addition to protein and carbohydrate. To be sure that P:C is influencing lifespan rather than some correlated component of yeast or another confounding change in diet composition will require using chemically defined diet formulations. No fully satisfactory such diet exists as yet for *Drosophila*, although Troen et al. [8] used four chemically defined diets in which the amino acid methionine and glucose were varied. Small but significant effects of dietary methionine on lifespan were reported.

However, chemically defined diets do exist for other insect species. It is well documented that lowering P:C in chemically defined diets slows the development of juvenile insects [9,10], and the recent work of Maklakov et al. [11,12] on adult crickets provides conclusive evidence that the ratio of protein to carbohydrate is the primary dietary determinant of lifespan in that insect (Figure 1C). Maklakov et al. fed field crickets, *Teleogryllus commodus*, one of 24 chemically defined diets and measured intake, lifespan, female lifetime egg production, daily egg production, male lifetime courtship singing effort, and singing effort per night. As for tephritids and *Drosophila*, crickets lived longest on low P:C ratio diets, and died progressively earlier as P:C ratio increased. Males but not females demonstrated a reduction in lifespan at high intakes of very low P:C diets; a result which was consistent with their greater propensity to lay down excess body fat on such diets and hence reflects the costs of obesity (a point that we consider further below). Again as for flies, female lifetime egg production was maximal at a higher P:C ratio than sustained maximal lifespan (Figure 1C). Male courtship singing attained a maximum at a lower P:C ratio than did female egg production.

The data for insects show that CR is not responsible for lifespan extension, rather, dietary P:C is critical: is the same true for mammals? It is widely held that CR, not specific nutrient effects, is responsible for lifespan extension in mammals [13,14]. However, we have argued previously [1] that it is not possible to estimate response surfaces such as those in Figure 1 without using a much larger number of diet treatments than have been employed to date in experiments on any mammal, including rodents. Without such surfaces it is simply not possible to separate CR from the effects of nutrient balance. Additionally, it has been reported over many years, notably in the early work of Morris Ross, that protein restriction, and of methionine in particular, extends lifespan in rodents [15-19]. Therefore, a study akin to that of Lee et al. [3] is required on rodents.

Whereas the experiments on insects have been able to concentrate on two macronutrient dimensions, protein and carbohydrate, a full design for rodents would need to extend to three dimensions by including variation in dietary lipid. An efficient initial design would need to include around 30 dietary treatments (e.g. 10 P:C:L ratios and 3 total concentrations), which would need to be fed to mice throughout their lives. This is challenging but by no means intractable - and would allow surfaces for lifespan and all manner of histological, biochemical and molecular variables, including those implicated in the process of aging, to be plotted onto macronutrient intake arrays.

To this point we have concentrated on evidence that increasing the ratio of protein and non-protein energy in the diet decreases lifespan; but as seen in the example from male crickets discussed above, if this ratio falls too far there is an increased risk of decreased longevity associated with obesity. The reason for this is that in omnivores and herbivores studied to date, protein intake is more strongly regulated than that of carbohydrate and fat [20]. As a result, protein appetite drives overconsumption of energy on low percent protein diets, promoting obesity and metabolic disorders with consequent effects on longevity. Overconsumption of energy on low percent protein diets has been reported for insects (e.g. [21]), fish (e.g. [22]), birds [23], rodents [24,25], nonhuman primates [26] and humans [20,27]. Fat deposition in response to excess ingested carbohydrate, driven by low dietary percent protein, has been shown to be labile in laboratory selection experiments in an  insect - it increased in response to habitual shortage of carbohydrate across successive generations and decreased in the face of persisting carbohydrate excess in the diet [28]. One adaptive mechanism that helps counteract the risk of developing obesity on low percent protein diets is increased facultative diet-induced thermogenesis, whereby excess ingested carbohydrates are removed via wastage metabolic cycles, e.g. involving uncoupling proteins [29].

In the context of the deleterious consequences of overconsumption it is interesting to note that the major causes of increased longevity in studies on calorically restricted primates (most recently [30]) is a reduction in the incidence of diabetes, cancer and cardiovascular disease relative to ad libitum fed controls. This may not result from benefits associated with CR per se, but rather reflect the costs of nutrient imbalance when feeding ad libitum on a fixed diet. As the required balance of nutrients changes over time (with time of day, season, growth and development, and senescence), animals will be forced to overeat some nutrients to gain enough of others. Even if a fixed diet is nutritionally balanced when integrated across the entire lifespan (and worse if it is not), changes in requirements at a finer timescale will result in accumulated damage from short-term nutrient excesses, which may be ameliorated by modest diet restriction [1].

When protein is eaten in higher then optimal quantities relative to non-protein energy it shortens lifespan - in insects certainly and perhaps too in mammals - but what might the underlying mechanisms be? There are several possibilities, including enhanced production of mitochondrial radical oxygen species [19,31], DNA and protein oxidative modification,  changes in membrane fatty acid composition and mitochondrial metabolism [19,32], changes in the relationship between insulin/IGF and amino acid signaling pathways, including TOR [33-38], toxic effects of nitrogenous breakdown products and capacity to deal with other dietary toxins [39,40], changes in immune function to pathogen attack [41,42], and changed functioning of circadian systems [43]. How these various components are interrelated will begin to emerge from analyses in which multiple biomarkers and response variables are mapped onto nutrient intake surfaces such as shown in Figure 1.

**Figure 2.**
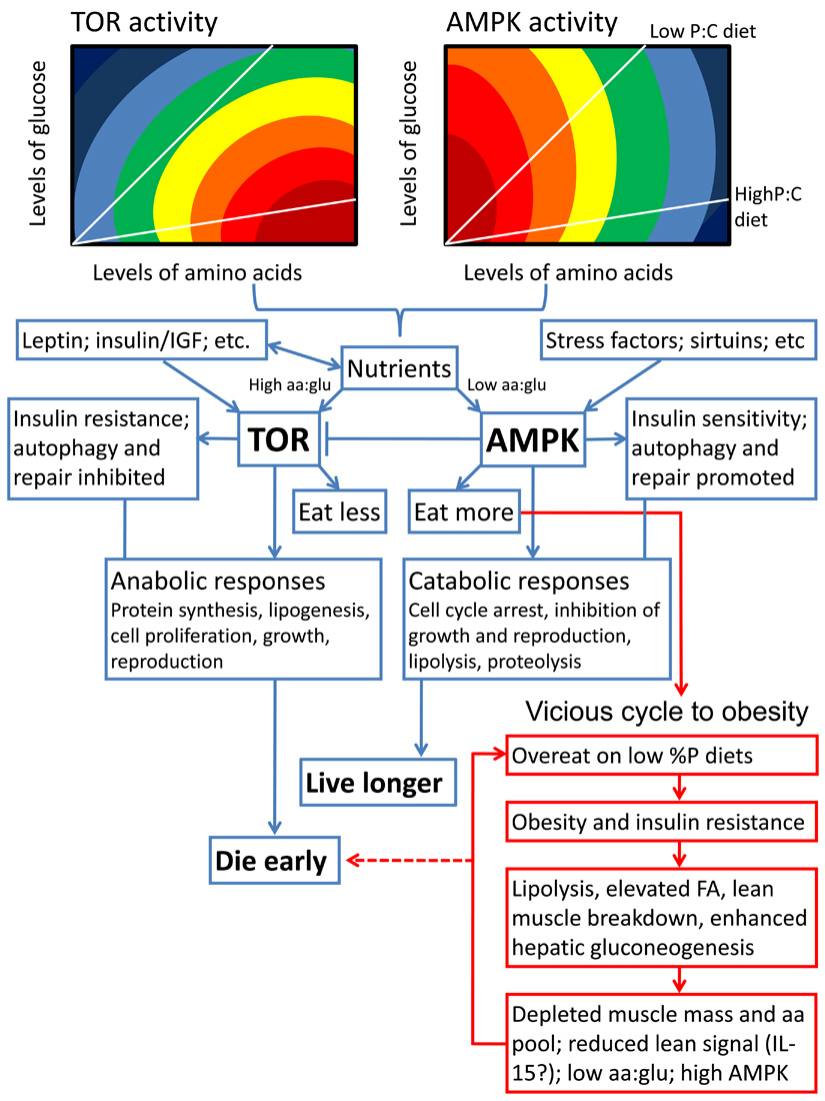
Schematic summarizing our hypothesis for how diet balance might affect lifespan via the TOR and AMPK signaling pathways. We propose that both TOR and AMPK respond not only to the concentration of circulating nutrients (with TOR activity stimulated and AMPK depressed either directly or indirectly by increasing concentrations), but also to nutrient balance. We show hypothetical response surfaces for TOR and AMPK in relation to circulating concentrations and ratios of amino acids (aa) and glucose (glu), with responses rising from dark blue to deep red. The red boxes indicate what we have termed the vicious cycle to obesity, in which chronic exposure to a low percent protein diet can drive overconsumption, metabolic disorders and shortened lifespan unless excess ingested energy is dissipated (see [20], and further supporting evidence from rodents in [52,53]). Otherwise, low percent protein diets are life extending via the normal actions of AMPK, whereas high percent protein diets shorten lifespan and encourage aging via the TOR pathway.

If we were to propose one candidate for the hub linking nutrient balance and other inputs to longevity it would be the interplay between the TOR and AMPK signaling pathways. Both TOR and AMPK serve as nutrient sensors and are linked to nutrient intake and metabolism. Factors that directly or indirectly increase TOR signaling, including elevated nutrients such a branch chain amino acids, glucose and fatty acids, are broadly anabolic and life-shortening. In contrast low levels of nutrients, declining ATP:AMP, and other influences that stimulate AMPK signaling are catabolic and life-extending [34; 38; 44-48] (Figure 2); - except when overconsumption, obesity and insulin resistance are driven by protein shortage on a habitually low percent protein diet [20] (see Figure 2). Although it is not yet establish whether TOR and AMPK are nutrient *balance* detectors, there are suggestions that they may well be. For example, glucose activates TOR in an amino acid-dependent manner [49] and elevated percent protein diet stimulates TOR and inhibits AMPK (e.g. [50,51]). We predict that mapping the responses of both TOR and AMPK onto nutrient intake arrays will provide fundamental new insights not only into aging, but also a whole range of interlinked metabolic phenomena, including obesity, type 2 diabetes, cancer risk and cardiovascular disease. To illustrate this point, we have predicted response surfaces in Figure 2 and linked aspects of nutrient balance, aging and obesity within a single schema.
